# The use of reproductive healthcare at commune health stations in a changing health system in Vietnam

**DOI:** 10.1186/1472-6963-11-237

**Published:** 2011-09-27

**Authors:** Anh D Ngo, Peter S Hill

**Affiliations:** 1 Social Epidemiology and Evaluation Research Group, School of Health Sciences, University of South Australia, City East Campus, Adelaide, SA 5000, Australia. (Formerly, Vietnam Evidence for Health Policy Project, School of Population Health, University of Queensland. Health Strategy and Policy Institute, 138 Giang Vo Str., Hanoi, Vietnam; 2 Vietnam Evidence for Health Policy Project, School of Population Health, University of Queensland, Herston Road, Herston 4006, Australia

## Abstract

**Background:**

With health sector reform in Vietnam moving towards greater pluralism, commune health stations (CHSs) have been subject to growing competition from private health services and increasing numbers of patients bypassing CHSs for higher-level health facilities. This study describes the pattern of reproductive health (RH) and family planning (FP) service utilization among women at CHSs and other health facilities, and explores socio-demographic determinants of RH service utilization at the CHS level.

**Methods:**

This study was based on a cross-sectional survey conducted in Thua Thien Hue and Vinh Long provinces, using a multi-stage cluster sampling technique. Questionnaire-based interviews with 978 ever-married women at reproductive age provided data on socio-demographic characteristics, current use of FP methods, history of RH service use, and the health facility attended for their most recent services. Multiple logistic regression analyses were used to identify socio-demographic determinants of their use of CHS RH services.

**Results:**

Eighty nine percent of ever-married women reported current use of birth control with 49% choosing intra-uterine device (IUD). Eighty nine percent of pregnant women attended facility-based antenatal care (ANC) with 62% having at least 3 check-ups during their latest pregnancy. Ninety one percent of mothers had their last delivery in a health facility. Seventy-one percent of respondents used CHS for IUD insertion, 55% for antenatal check-ups, and 77% gynecological examination. District and provincial/central hospitals dominated the provision of delivery service, used by 57% of mothers for their latest delivery. The percentage of women opting for private ANC services was reported at 35%, though the use of private delivery services was low (11%). Women who were farmers, earning a lower income, having more than 2 children, and living in a rural area were more likely than others to use ANC, delivery, and/or gynecological check-up services at the CHS.

**Conclusions:**

Women choice of providers for FP and RH services that help them plan and protect their pregnancies is driven by socio-economic factors. While the CHS retains significant utilization rates, it is under challenge by preferences for hospital-based delivery and the growing use of private ANC services.

## Background

With the introduction of its "*Doi Moi*" or 'renovation' policy in 1986, Vietnam has shifted from a centrally planned towards a market-oriented economy. Health sector reform has introduced user fees in public hospitals and legalized private practice. In urban centers in particular, the private health sector has been growing rapidly, and increasingly is in competition with the public sector in providing health services. These changes have produced profound effects on the use of health services at state health facilities, including RH care at the local CHS - the basic unit of the primary healthcare system in Vietnam [1,2].

To date, no study has examined the use of FP and primary RH services in the context of the current healthcare system in Vietnam, as it shifts from a state monopoly to a more pluralist system. To address this shortcoming, this study aims to describe the pattern of FP and RH service utilization among women at the CHS and other state and private facilities, and to explore socio-demographic determinants of RH service utilization at the CHS level. It is intended to inform policy and program reviews that will strengthen the CHS primary RH network in the changing health system of Vietnam.

The state health system in Vietnam consists of 4 levels: the national level with the Ministry of Health and central hospitals; the provincial level with the Provincial Department of Health and provincial hospitals; the district level with district health centers and district hospitals; and the communal level with CHSs. RH and FP services are available at all-level state health facilities: central, provincial, district hospitals and CHSs.

Despite the changes in the health sector, the CHS retains its responsibilities as the primary access point to primary RH and FP services subsidized by the state budget, and is required to meet government numerical targets on basic RH and FP service use indicators. Every CHS is staffed by at least one midwife who acts as the focal point for RH services, including antenatal and post-natal care, delivery, gynecological examination and treatment, and FP services such as IUD, oral contraceptives, and condoms. Some CHSs located close to a hospital are restricted from providing delivery services. Local residents are entitled to free services at their local CHS but not at the CHS in other communes.

In addition to everyday RH services, CHSs are entrusted with responsibilities to register and manage all pregnant women in the commune, conducting 1-day monthly antenatal check-ups. There are also 3-day semi-annual RH campaigns based at the CHS with assistance of midwifes or doctors from the district hospital. The primary purpose of these campaigns is early detection and treatment of reproductive tract infections (RTIs) including sexually transmitted infections, based on clinical examination [3]. Since the restructuring of the national population program, there is a network of population collaborators affiliated with the local CHS, who have basic training in RH health education and services. Population collaborators conduct regular outreach communication activities, providing RH counseling and contraceptives (e.g. condoms, pills), and referring couples to the CHS for further FP and RH services such as IUD insertion, gynecological and antenatal check-ups [4].

Private healthcare services in Vietnam comprise three differing service structures [5,6]. Private hospital care is principally located in major cities. Compared to state hospitals, they have a reputation for their access to more advanced diagnostic and therapeutic technology, and their capacity to provide technically demanding services. Private outpatient clinics are operated by individuals or groups of full- or part-time physicians in urban, sub-urban and rural areas, under a license from provincial health authorities. They largely provide general or specialized health services for middle income patients. Third, "mobile" private practitioners--who can be nurses, assistant doctors, or retired doctors--are commonly present in rural areas, providing health services from their own homes without a license. These health practitioners are not included as part of the formal health system, although they play an important role in providing basic health services [6]. For RH services at the commune level, private providers include doctors or practitioners (e.g. midwives, assistant doctors), and traditional birth attendants, who provide home-based delivery services [7,8]. In addition, local pharmacies can offer the commercial sale of birth control commodities such as oral contraceptives, without prescription.

With the reduction in government subsidies for CHS facilities, equipment, and human resources development, the quality of health services at CHSs has been perceived as deteriorating and is associated with less-qualified health staff, outdated equipment, limited drugs and supplies [2]. At the same time, the private sector has been increasingly popular and is associated with convenient opening hours, more competent staff, better equipment, and effective drug prescriptions [9]. Reports suggest that increasingly, patients are seeking care at private clinics or bypassing their local CHS for higher level health facilities, willing to pay higher fees based on their perceptions of better quality and enhanced technology [1,2,10,11].

### The Study Sites

This study is part of the baseline assessment of a project supported by Marie Stopes International Vietnam, that aims to create public-private partnerships to improve access to services and the quality of primary RH care, in two provinces, Thua Thien Hue and Vinh Long, in Vietnam. Thua Thien Hue is located in the center of Viet Nam with a total population of 1,143,500, of which 284,149 women are at reproductive age (15 - 49). The province has 1 central hospital, 9 district hospitals, and 152 CHSs located in urban, semi-urban, rural, and mountainous areas. Based in the Mekong delta region, Vinh Long province has a population of 1,057,000. The state health care network consists of 107 CHSs, 8 district hospitals, and 1 provincial hospital. People in districts bordering with Can Tho city may seek RH services at the Can Tho central hospital. Almost all CHSs in the 2 provinces are staffed by a doctor [4].

Studies sites included 1 urban district (Hue city), 2 rural districts of Thua Thien Hue, and 3 rural districts of Vinh Long. As these districts received no external support for primary RH care, they can be considered to represent the baseline for state provided RH services. In each of these districts, the state primary healthcare network was well-established, with each commune having a CHS and a team of population collaborators. In contrast, RH private clinics were mostly concentrated in urban areas, and were often run by a gynecologist working in a major hospital. Only 3 out of 5 rural districts had a gynecologist working in the district hospital and 2 rural districts in Vinh Long had an accredited private RH clinic (i.e. with a license from provincial health authorities) operated by this specialist [12].

## Methods

The study was a cross-sectional survey, using a multi-stage cluster sampling design to recruit participants. The first stage selected 5 communes from each of the selected districts, using proportional to population size techniques, totaling 30 communes in the 2 provinces. The second stage involved the random selection of 1 village or residential group in each commune where 30 households were randomly drawn for interviews of all women at reproductive age (15-49). In total, 900 households with 1 417 women were included in this study.

Data was collected using questionnaire-based interviews, consisting of mutiple-choice questions on respondents' socio-demographic characteristics (e.g. age, marital status, occupation, education, number of living children, income), their RH and FP service use (see Additional File 1). Women were also asked about the health facility where they attended antenatal care during their last pregnancy or received their most recent gynecological examination, and the place where they had their latest delivery. These three RH services were considered to constitute basic RH healthcare provided at the local CHS or other state health facilities in Vietnam. Women were interviewed at home by a trained interviewer who was a member of the commune Women's Union. Data collection was completed from April to May, 2010. As mentioned earlier, the research was undertaken as a baseline survey, commissioned by Marie Stopes International Vietnam for planning an intervention in the 2 selected provinces. Ethical approval was given by the Ethic Committee of the Thua Thien Hue and Vinh Long Provincial Departments of Health.

Data were entered into Epi Info, then transferred to STATA version 9.0 for processing and analyses. Ten percent of questionnaires were double entered to check consistency. Data analyses consisted of two sequential steps. First, descriptive analysis was performed of the socio-demographic characteristics of all study participants and the frequency distributions of variables of interest, including the current use of FP method(s), history of ANC, delivery, and gynecological check-up service use, and the health facility where women attended for their most recent service.

Second, logistic regression analyses were performed of factors related to the use of the following services in the local CHS: (i) antenatal check ups during the last pregnancy, (ii) their latest delivery, and (iii) their most recent gynecological examination. The dependent variable was based on whether or not women who visited a health facility for these services reported selecting their local CHS. Independent variables of interests included: women's age (above vs. below the median), number of living children (having 2 children or less vs. 3 children or more), religion (Buddhist, Catholic, other, and none), ethnicity (majority vs. minority), occupation (farming vs. other), education (secondary school or less vs. high school or more), income (4 quartile groups), current living location (urban vs. rural), and study province (Vinh Long vs. Thua Thien Hue). The statistical model for the use of CHS delivery services excluded those who required a cesarean section and those who reported that delivery service was unavailable at their local CHS at the time the women gave birth. Each socio-demographic variable was analyzed first as a univariate predictor to obtain a crude odds ratio (OR) and 95% confidence intervals (CI). Significantly related variables (*p <*0.05) were subsequently included in a multivariate analysis to obtain an adjusted OR and 95% CI. Only factors that were significantly associated with the outcome variable in the multi-variate analytical model were retained in the final reduced model.

## Results

The sample consisted of 678 women (48%) in Vinh Long and 739 women (52%) in Thua Thien Hue. Median age was 31. Sixty-six percent of respondents were married; 31% were single, and 3% were widowed or divorced. The demographic characteristics of respondents are presented in Table 1.

**Table 1:** Socio-demographic characteristics of surveyed respondents (n = 1417)

Variable	n	%	Variable	n	%
**Province**					

Vinh Long	678	48	**Religion**		

Thua Thien Hue	739	52	Buddhist	556	39

**Age**			Catholic	75	5

< = 31	740	52	Other	58	4

> 31	677	48	None	728	52

**Marital status**			**Occupation**		

Single	439	31	Farmer	570	40

Ever-married	978	69	Non-farming job	847	60

**Number of children**			**Location of living**		

None	435	33	Urban	213	15

1 or 2	635	45	Rural	1204	85

> 2	307	32	**Income**		

**Education**			1st quartile	356	25

Secondary school or less	960	68	2nd quartile	416	29

High school or more	457	32	3rd quartile	296	21

			4th quartile	349	25

### FP service use

Among 439 single women, only 13 (3%) reported having a current sexual partner, and 9 reported current use of a birth control method. Given these small numbers, analyses of FP service use were limited to ever-married participants (n = 978). The rate of FP service use among ever-married women was 88.5% after excluding those who reported not using a birth control due to one of the following reasons: being pregnant, having just given birth, or being menopausal. IUD appears to be the most common method, utilized by 49% of women, followed by condoms (16%), oral contraceptives (15%), and the rhythm method (14%). Male or female sterilization was used by 5.5%, and injected contraceptives by 3.8% of married women. The sum of these percentages was 104% as a few women reported concurrent use of condoms and the rhythm method.

Regarding the source of FP services, 71% of the 464 women using IUDs reported visiting the local CHS, with 19% selecting district hospitals, 8% private clinics, and 2% other facilities for their last IUD insertion. For condoms and other contraceptives (e.g. oral, injections), the CHS appears to be the major source, reported by 61% women, followed by population collaborators (37%), and then pharmacies (18%). Only 5% of women had obtained these birth control supplies from a hospital, with 2% using a private clinic (Table 2). The total percentage of 123% reflects the availability of contraceptives from multiple sources.

**Table 2:** Sources of FP services

Health facility	Last IUD insertion (n = 464)		Condoms/other contraceptives	
	**n**	**%**	**n**	**%**

CHS	329	71.0	345	60.8

Pop. Collaborators	NA	NA	208	36.7

Pharmacy	NA	NA	102	18.0

Dist. Hospital	86	18.5	24	4.2

Provincial/central hospital hospital	8	1.7	5	0.9

Private clinics	37	8.0	11	1.9

Other	4	0.9	11	1.9

NA: Not applicable

### Antenatal care service use

Of 964 women who had ever experienced a pregnancy, 857 (89%) reported having facility-based antenatal care with 62% having at least 3 check-ups during their last pregnancy. Among these 857 women, more than half (55%) used the CHS, 35% private clinics, and 31% the district hospital. Approximately 10% of women went to the provincial or central hospital for this service. The total percentage was greater than 100%, as women may visit multiple health facilities during the gestational period (Table 3).

**Table 3:** The use of antenatal check-up and delivery service by health facility

Health Facility	Antenatal check-up (n = 857)		Delivery (n = 942)		Gyn. examination (n = 759)	
	
	n	%	n	%	n	%
CHS	473	55.2	181	19.2	585	77.1

Dist. hospital	269	31.4	351	37.3	62	8.2

Prov./Cent. hospital	84	9.8	188	20.0	24	3.2

Private clinic	303	35.4	106	11.3	80	10.5

Home	NA	NA	66	7.0	NA	NA

Self-delivery	NA	NA	22	2.2	NA	NA

Other	6	0.7	28	3.0	8	1.0

NA: Not applicable

**Figure 1:**
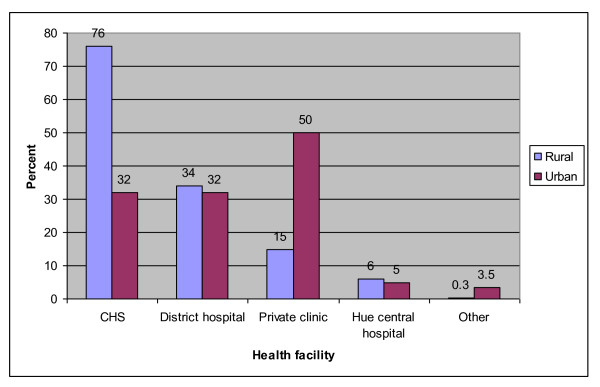
**Health facility visited for antenatal check-ups at the latest pregnancy in Thua Thien Hue (n = 402)**.

**Figure 2:**
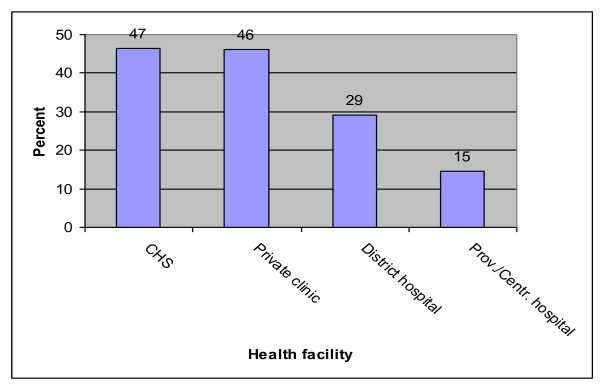
**Health facility visited for antenatal check-ups at the latest pregnancy in Vinh Long (n = 455)**.

Analyses of data by provinces found that in Thua Thien Hue, the local CHS was the most favorable service provider in rural districts, attracting 76% of women, compared to district hospitals (34%), and private clinics (15%). In the city, private clinics ranked first, visited by 50% of women, with the local CHS and district hospital each reporting access by 32% of women. In both settings, around 5% of women reported visiting Hue central hospital for ANC services during their last pregnancy (Figure 1). In Vinh Long where the survey only included rural communes, an almost equal proportion of women (47% and 46%) reported using CHSs and private clinics, with 29% visiting the district hospital, and 15% going to the provincial or central hospital for ANC services (Figure 2).

### Delivery service use

Of 942 women who reported a delivery, 854 (91%) had their latest delivery in a health facility. The district hospital was selected by the highest proportion of women (37%), followed by provincial or central hospitals (20%), then CHS(19%), and private providers(11%). Only 7% of women had home-based delivery with a birth attendant and 2% practised self-delivery (Table 3).

### Gynecological check-up service use

Sixty four percent of surveyed women (n = 759) reported having undertaken gynecological check-ups. During the most recent check-up, the highest percentage (77%) visited the local CHS, 11% opted for private clinics, 8% went to the district hospital. Only 3% went to the provincial/central hospital for this service (Table 3).

### Socio-demographic determinants of RH service use at the local CHS

Table 4 presents the results of the logistic regression analyses. The analyses found that the CHS antenatal check-up and gynecological examination services were both strongly related to number of children (< = 2 vs. > 2), religion (Buddhist vs. each of other groups: Catholic, non-Buddhist and non-Catholic, and non- religious affiliation), occupation (farmers vs. other groups: government cadres, factory workers, small traders, and students), and self-reported income (first quartile vs. each of other 3 quartiles). Women with more than 2 children, having a non- Buddhist/non-religious affiliation, or living in Thua Thien Hue (compared to Vinh Long) were more likely than others to attend antenatal check-ups during their latest pregnancy, or to have their most recent gynecological examination at their local CHS. Women with a non-farming occupation or earning a higher income were less likely than others to visit their local CHS for these two services. Regarding delivery service use, mothers of minority ethnic groups were more likely than those of the majority ethnic group to have their latest delivery at the local CHS, which was also true for non-Buddhist/non-religious mothers compared with Buddhist mothers. Women from non-farming occupations, or earning a higher income, were less likely to choose CHS-based delivery. It is noted that the use of CHS antenatal check-up service was significantly related to living location, with rural women more likely than their urban counterparts to use this CHS service. None of women in the city reported having their latest delivery at the CHS.

**Table 4:** Factors related to the use of RH services available at the local CHS (Reduced logistic regression model)

Factors	Category	OR (95% CI)		
		**Antenatal check-up (n = 835)**	**Delivery** **(n = 577)**	**Gynecological check-up (n = 718)**

**Children**	< = 2	Ref	Ref	Ref

	> 2	1.61(1.13-2.31)	NS	1.62(1.01-2.06)

**Religion**	Buddhist	Ref	Ref	Ref

	Catholic	2.91(1.36-6.25)	3.05(1.49-6.26)	3.19(1.04-9.85)

	Other	3.10(1.39-6.90)	2.85(1.16-7.01)	7.91(2.24-27.97)

	None	2.01(1.38-2.92)	2.17(1.39-3.38)	3.60(2.36-5.49)

**Ethnicity**	Majority	Ref	Ref	Ref

	Minority	NS	2.20(1.32-3.69)	NS

**Occupation**	Farming	Ref	Ref	Ref

	Non-farming	0.64(0.45-0.90)	0.22(0.15-033)	0.72(0.57-0.91)

**Living location**	Urban	Ref	Ref	Ref

	Rural	2.79(1.59-4.91)	ND	NS

**Province**	Vinh Long	Ref	Ref	Ref

	Thua Thien Hue	2.83(1.88-4.26)	NS	3.33(2.11-5.26)

**Income**	1st quartile	Ref	Ref	Ref

	2nd quartile	0.79(0.53-1.17)	0.73(0.47-1.13)	0.41(0.24-0.71)

	3rd quartile	0.52(0.33-0.83)	0.57(0.34-0.97)	0.40(0.22-0.75)

	4th quartile	0.42(0.26-0.67)	0.33(0.18-0.61)	0.34(0.18-0.63)

NS: not statistically significant so not included in the model; ND: the variable is excluded because none of the urban women reported delivery at CHSs

## Discussion

This study is among the first examining the pattern of RH and FP service utilization at the CHS and in other health facilities in the context of change in the healthcare system in Vietnam. With basic RH services available from a range of health facilities, women in Vietnam, especially those in urbanized areas, have been given a range of alternatives. However, state services continue to dominate, though private services are playing a significant role. In general, the local CHS appears to be the most frequently used health facility for FP, ANC, and gynecological examination services, all free or heavily subsidized. Hospitals dominate the provision of delivery services. There were marked differentials in women's selection of service providers for ANC and delivery services between rural and urban areas.

Data on behavioral RH care indicators in the selected districts were impressive with a very high proportion of married women currently using a birth control method, high rates of pregnant women having sufficient ANC and giving birth at a health facility. These data were comparable with the national-level statistics over the past 5 years [13] that reported high national rates of contraceptive use (80%-90%), at least 3 antenatal visits in pregnant women (87%), and deliveries attended by trained health personnel (95% or higher) [14,15]. The pattern of birth control methods currently used was also keeping with the national data that reported IUD to be the most popular method applied by around 56% of married couples [14].

The high levels of contraceptive use, with the local CHS as the major supplier, indicates that the model of providing FP services in Vietnam is effective, and able to meet local needs. Historically, the CHS has been serving as the primary access point for FP services under the national population and family planning program since early 1960s. Over time, the CHS has gained a reputation for its provision of subsidized FP services, with delivery of FP services strengthened by the population collaborator network, providing women with easy access to low-cost, community-based services. The contribution of the local pharmacy is also significant, diversifying the provision of condoms or other contraceptive commodities at the commune level. The hospital and private providers appear to have a little role in the provision of IUD services and other FP commodities (i.e. condoms or pills).

The CHS also appears to be a major provider for antenatal check-ups, particularly in the rural area, although the number of private antenatal service users was significant. This finding counters previous studies that reported lower use of primary healthcare and outpatient treatment services at CHSs compared to private clinics or hospitals [1,16]. Antenatal care check-ups at the CHS are subsidized and accompanied by free pre-natal tetanus vaccination, and the CHS is proactive in inviting mothers to register for the monthly antenatal clinic when their pregnancy is confirmed. However, while CHSs continue to dominate the provision of ANC services, a preference for delivery in hospitals was clear in these districts, a finding consistent with rising economic status and increasing patient expectations of the health system in Vietnam [2,8,17].

Living location affects the selection of ANC service providers, with rural women more likely than their urban counterparts to use the CHS. The same differential was not seen in the choice of services relating to delivery, with women living in rural areas as likely to choose a hospital as those in the city. In both settings, district hospitals have become the most popular provider for this service, while the role of the local CHS has tended to diminish. Our data support earlier studies that found a low use of CHS delivery services, with most women preferring district hospital-based delivery because of the perceived better quality of services for both mothers and newborn [7,8]. The data also support the qualitative study in these provinces that noted the 2-child population control policy has made couples more cautious about outcomes for their baby, choosing hospital-based delivery where they are more confident in provider's expertise and equipment, and life saving emergency care is readily available [4].

The high use of gynecological check-up services at the local CHS was attributed in part to the national RH semi-annual campaign. Although this national campaign is aimed at early detection and treatment of RTIs, population-based studies conducted within the last 10 years, including one study in Thua Thien Hue, reported a prevalence of RTIs ranging from 21% to 39% among women of reproductive age [18-20]. The RTIs prevalence was found to be even higher in hospital- or clinic-based studies, though these were subject to selection bias [21,22]. While RTIs can change overtime and vary by provinces, such high prevalence raises questions around the strategic use of CHS-based free gynecological check-ups during the national semi-annual RH campaign, with questions over the quality and efficacy of this service as an intervention strategy.

Private physicians were important providers for ANC services in both rural and urban areas, despite their uneven geographical distribution. With RH private clinics mostly concentrated in urban areas, using private services not only incurred higher fees, but also travel expenses for rural women. Yet, a significant proportion of rural women used private clinics during their latest pregnancy. Previous studies report criticism of state facilities and a preference for reproductive healthcare provided at private clinics, though, paradoxically, these private clinics are serviced by physicians who routinely work in state health facilities [23]. In the private clinics, clients may be entitled to direct access to more senior clinicians, and find staff more responsive, compared to their reception in state services [23]. While CHSs maintained dominance in provision of ANC services, there was a clear upwards trend for the use of private antenatal services, consistent with trends for other health service use in Vietnam [1,2].

In essence, the patterns of RH service provider choice were driven by socio-economic and geographic factors. Women of lower socio-economic status, who were farmers, earning a lower income, having more than 2 children, and living in a rural area were more likely to use antenatal, delivery, and/or gynecological check-up services at the local CHS. This finding is consistent with previous studies that found the poor tend to use the CHS more frequently than the rich [1,24].

Among the 2 provinces, a higher percentage of women in Thua Thien Hue reported visiting their local CHS for ANC and gynecological check up services. This difference can be explained by the higher number of CHSs in sampled communes in Thua Thien Hue staffed by a doctor (13), compared to those in Vinh Long (10) [25]. In Vinh Long, 2 of 3 districts included in the survey had an accredited private RH clinic, while there were none in rural districts of Thua Thien Hue. This was reflected in the findings that more rural women in Vinh Long used antenatal check-ups at private clinics and fewer of them used this service at the CHS compared to their counterparts in Thua Thien Hue, though in both provinces, there were low levels of delivery in private facilities. This difference also indicates a shift from CHSs to private doctors for ANC services where they are locally accessible, reducing the use for services delivered through the CHS system.

The study findings should be considered in conjunction with the limitations of the research. The sample was drawn from districts purposively selected for the intervention project, and thus not representative for the whole province. Although the percentage of women who have a current sexual partner (3%) was consistent with national data on prevalence of premarital sex among young women (5.2%), [26] the sample of unmarried women was too small for meaningful statistical analyses. Data were self-reported, and may incur recall bias with regards to the history of RH service use, particularly with ANC and delivery services.

## Conclusions

Although the CHS in this study retains significant utilization rates and constitutes an important provider of primary RH services, it is under challenge on three fronts. The first challenge is from the significant use of private practice for ANC, where clients have direct access to higher grades of health care providers, and are willing to pay higher fees for their perceptions of better quality of care. Secondly, although the semi-annual gynecological screening continues to attract significant numbers of women with its subsidized service, its effectiveness as a population strategy is questionable, and the quality of services needs evaluation. Thirdly, the preference for delivery in district hospitals over CHS is marked, and arguably, government services would do well to move towards strengthened referral services and shared care with the CHS, rather than invest in duplication of these functions at both levels.

To address these challenges, the CHS system needs to be responsive to specific local needs. In areas with accessible alternative health services reducing the need for CHS services, rationalizing of services is needed. Those CHSs that attempt to function in the shadow of hospital facilities need to be protected by referral practices and clear differentiation of services to avoid unnecessary provision of routine RH care by more costly staff, in higher cost level facilities. At the same time, efforts should be made to improve service quality at the local CHS as the economy improves and clients demonstrate growing demand for higher quality services. With the reduction in government subsidies, a responsive payment scheme must be developed at the CHS level that generates income for the sustainable provision of quality services, but retains subsidized services for the poor who continue to rely on their local CHS for basic reproductive healthcare.

## Abbreviations

ANC: Antenatal care; CHS: Commune Health Station; CI: Confidence Interval; FP: Family Planning; IUD: Intra uterine device; RH: Reproductive Health; OR: Odd Ratios

## Competing interests

The authors declare that they have no competing interests.

## Authors' contributions

AN conceived research ideas, developed research protocol and data collection tools, and conducted data collection and data entry. AN performed data analysis and drafted the manuscript with input from PH. PH made a substantial contribution in revising the manuscript for intellectual content. All authors reviewed and approved the final version.

## Pre-publication history

The pre-publication history for this paper can be accessed here:

http://www.biomedcentral.com/1472-6963/11/237/prepub

## Supplementary Material

Additional file 1 **Study questionnaire**. Click here for file
