# Supplementary material for: The use of reproductive healthcare at commune health stations in a changing health system in Vietnam
Source: BMC Health Serv Res. 2011 Sep 27;11:237. doi: 10.1186/1472-6963-11-237 (PMC3189878; doi:10.1186/1472-6963-11-237)
Supplement: Additional file 1 — Study questionnaire. [file 1472-6963-11-237-S1.DOC]

**QUESTIONNAIRE**

*Screening question:*

In what year were you born? **19....**

**Before 1961 or after 1995, thank the respondent and end the conversation**

| **No** | **Questions** | **Coding anwsers** | **Skip** |
| --- | --- | --- | --- |
| **THE USE OF FAMILY PLANNING SERVICES** | | | |
| Q201 | What birth control method(s) you are currently using ? | 99. None   1. IUD 2. Oral contraceptives 3. Injectable/inplant contraceptives 4. Condom 5. Rhythm method 6. Male sterilization (for husband) 7. Female sterilization 8. Other, specify..................... | Q201a |
| Q201a | If none, why ? | 1. Currently not having a sexual partner 2. Don’t know about FP method 3. Being pregnant 4. Not having sex due to having just given birth 5. Husband/partner disagrees 6. Unable to obtain services in the living location 7. Wanting to have a son 8. Other/specify.................................... | Q206 |
| Q202 | Where do you often obtain contraceptive pills or condoms? | 99. Never   1. District hospital 2. Provincial/central hospital 3. Provincial Center for RH care 4. Private clinic 5. Pharmacy 6. Population collaborator 7. Local market 8. Other, specify................................ |  |
| Q204 | Where (in which health facility) did you have the most recent IUD insertion? | 99. Never have an IUD insertion   1. Commune health station (CHS) 2. District hospital 3. Provincial/Central hospital 4. Provincial Center for RH care 5. Private clinic   6. Other, specify...................... |  |
| Q205 | How many living children do you have ? | 1. Son...................  2. Daughter.........................  99. None |  |
| **THE USE OF ANTENATAL CARE (ANC) SERVICES** | | | |
| Q206 | When did you have the last pregnancy ? | Year ...............  Don’t remember when  99. Not yet | Q218 |
| Q207 | How many ANC visits did you have in the last pregnancy? | ……….....visits  99. None |  |
| Q207a | If none, Why ? | 1. Don’t know about ANC 2. Feel unecessary 3. Services far from home 4. Be concerned about service fees 5. Other, specify .................. | Q213 |
| Q208 | Where (in which health facility) did you have ANC check ups in your last pregnancy? | 1. Local CHS 2. District hospital 3. Provincial/Central hospital 4. Provincial Center for RH care 5. Private clinic 6. Other, specify............... | Q213 |
| Q209 | Why you did not go to the local CHS for ANC services ? | 1. Low levels of expertise 2. Inadequate equipment 3. Inadequate medicine 4. Poor facilities (room, bed) 5. Unfriendly staff 6. Far from home 7. Service unavailable at the CHS 8. Lack of ultrasound service 9. Other, specify…………… |  |
| **THE USE OF DELIVERY SERVICE** | | | |
| Q213 | When did you have your last delivery ? | ....month.....................year................  88. Don’t remember when  99. Not yet having a delivery | Q218 |
| Q214 | Where did you have your last delivery? | 1. Local CHS 2. District hospital 3. Provincial/Central hospital 4. Provincial Center for RH care 5. Private clinic 6. At home with a birth attendant 7. Self-delivery 8. Other,specify.............................. | Q216 |
| Q215 | Why you did not have your last delivery at your local CHS? | 1. Low level of expertise 2. Inadequate equipment 3. Inadequate medicine 4. Poor facilities (room, bed) 5. Unfriendly staff 6. Far from home 7. Service unavailable at the CHS 8. Having a cesarean   9. Other, specify…………… |  |
| **THE USE OF GYNECOLOGICAL CHECK UP SERVICES** | | | |
| C218 | When did you have your most recent gynecological check up? | month.....................year.................  99. Never having a check up  88. Don’t remember when | C502 |
| C219 | The reason for undergoing this check up | 1. Experiencing a symptom 2. Being invited by health staff during a RH campaign 3. Going for a regular check up 4. Other,specify.............................. |  |
| C220 | Which health facility did you go for this check up? | 1. Local CHS  2. District hospital  4. Provincial/Central hospital  5. Provincial Center for RH care  6. Private clinic  7. Other, specify............... | C502 |
| C221 | Why you did not go to the local CHS for this check up? | 1. Low levels of expertise 2. Inadequate equipment 3. Inadequate medicine 4. Poor facilities (room, bed) 5. Unfriendly staff attitudes 6. Far from home 7. Service unavailable at the CHS   8. Other, specify…………… |  |

**BACKGROUND INFORMATION**

| **No.** | **Questions** | **Coding of answers** | **Skip** |
| --- | --- | --- | --- |
| Q502 | What is your marital status? | Single 1  Married 2  Divorced 3  Separated 4  Widowed 5 |  |
| Q503 | What religion are you? | Buddhist 1  Protestant 2  Catholic 3  Ancestral worship 4  No religion 5  Other religion (describe) 6  ………………………………… |  |
| Q504 | To which ethnic group do you belong? | Kinh 1  Other ethnicity (describe) 2  ………………………………….. |  |
| Q505 | What is the highest level of education you have finished? | University/college 1  Vocational training 2  High school (Grade 10 – 12) 4  Secondary school (Grade 6-9) 5  Primary school (Grade 1-5) 6  Illiterate 7 |  |
| Q506 | What do you do to earn a living now | Farmer 1  Teacher/government cadre 3  Factory worker 4  Small trader 5  Students 6  Other/specify……………... 88 |  |
| Q507 | What is your average monthly income? | .....................million VND |  |
| Q508 | What is the location of the commune | Urban 1  Rural 2 |  |

**Thank the respondent and end the interview!**
